# Structural and Thermodynamic Analysis of the Resistance Development to Pimodivir (VX-787), the Clinical Inhibitor of Cap Binding to PB2 Subunit of Influenza A Polymerase †

**DOI:** 10.3390/molecules26041007

**Published:** 2021-02-14

**Authors:** Jiří Gregor, Kateřina Radilová, Jiří Brynda, Jindřich Fanfrlík, Jan Konvalinka, Milan Kožíšek

**Affiliations:** 1 Institute of Organic Chemistry and Biochemistry of the Czech Academy of Sciences, Gilead Sciences and IOCB Research Center, Flemingovo n. 2, 16610 Prague 6, Czech Republic; jiri.gregor@uochb.cas.cz (J.G.); katerina.radilova@uochb.cas.cz (K.R.); jiri.brynda@uochb.cas.cz (J.B.); jindrich.fanfrlik@marge.uochb.cas.cz (J.F.); 2 First Faculty of Medicine, Charles University, Kateřinská 1660/32, 12108 Prague 2, Czech Republic; 3 Department of Biochemistry, Faculty of Science, Charles University, Hlavova 8, 12800 Prague 2, Czech Republic

**Keywords:** influenza A polymerase, antivirals, pimodivir, VX-787, resistance

## Abstract

Influenza A virus (IAV) encodes a polymerase composed of three subunits: PA, with endonuclease activity, PB1 with polymerase activity and PB2 with host RNA five-prime cap binding site. Their cooperation and stepwise activation include a process called cap-snatching, which is a crucial step in the IAV life cycle. Reproduction of IAV can be blocked by disrupting the interaction between the PB2 domain and the five-prime cap. An inhibitor of this interaction called pimodivir (VX-787) recently entered the third phase of clinical trial; however, several mutations in PB2 that cause resistance to pimodivir were observed. First major mutation, F404Y, causing resistance was identified during preclinical testing, next the mutation M431I was identified in patients during the second phase of clinical trials. The mutation H357N was identified during testing of IAV strains at Centers for Disease Control and Prevention. We set out to provide a structural and thermodynamic analysis of the interactions between cap-binding domain of PB2 wild-type and PB2 variants bearing these mutations and pimodivir. Here we present four crystal structures of PB2-WT, PB2-F404Y, PB2-M431I and PB2-H357N in complex with pimodivir. We have thermodynamically analysed all PB2 variants and proposed the effect of these mutations on thermodynamic parameters of these interactions and pimodivir resistance development. These data will contribute to understanding the effect of these missense mutations to the resistance development and help to design next generation inhibitors.

## 1. Introduction

Influenza viruses cause lung and upper respiratory tract infections. There are four types of the influenza virus–A, B, C and D. Influenza virus A, B and C cause respiratory disease in humans, while the influenza D virus infection has not been observed in humans. Influenza A virus (IAV) carries the pandemic potential and is dangerous especially for children, elderly, chronically ill and immunodeficient people [1,2]. Due to the boundless and extensive spread of IAV, it has a major impact on humanity, health of population, pandemic potential, resistance to nowadays drugs but also considerable impact on economy. Therefore, it is necessary to keep looking for novel anti-influenza drugs [3].

**Figure 1:**
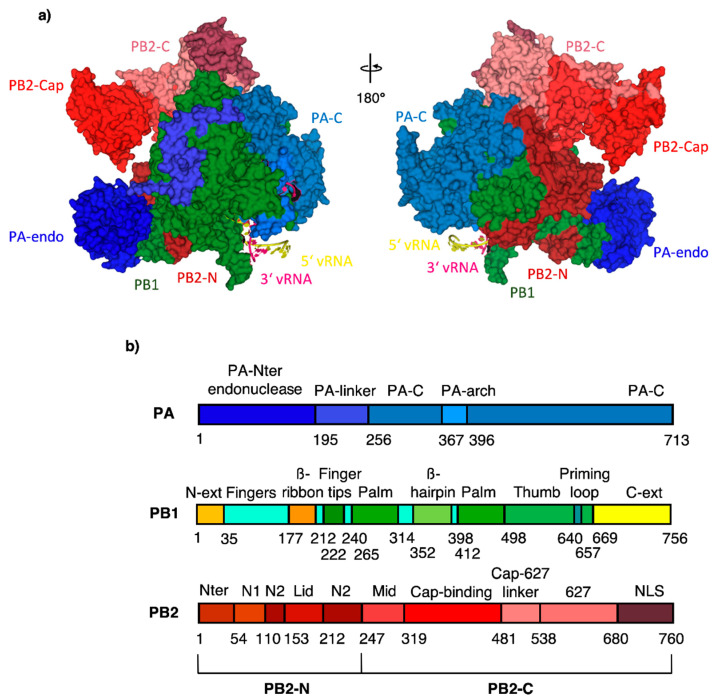
(**a**) RdRp subunit domain structure with subdomain names; color-coding according to the domain structure viewed in (**b**) except PB1 and PB2-N, which are uniformly in green and dark red for clarity. PDB code 4WSB [5].

One of the IAV drug targets is the RNA-dependent RNA polymerase (RdRp). RdRp of IAV is highly conserved among influenza viruses [4]. It is composed of three subunits–the polymerase acidic protein (PA) with endonuclease activity, the polymerase basic protein 1 (PB1) with polymerase activity and the polymerase basic protein 2 (PB2) containing five-prime cap binding site (CBS) (Figure 1) [5]. IAV does not encode guanylyl transferase, hence no cap is bound to viral mRNA and proteins cannot be translated using host cell machinery. Moreover, without a cap-bound primer achieved from the host cell, transcription cannot occur. It is thus necessary for the virus to “steal” the host five-prime cap in a crucial process called cap-snatching. This process is enabled by organized cooperation of all three subunits of RdRp. Host five-prime cap binds to the PB2 cap-binding site, then PA cleaves 10–13 nucleotides from the host five-prime cap and PB1 synthesizes viral RNA [6,7].

**Figure 2:**
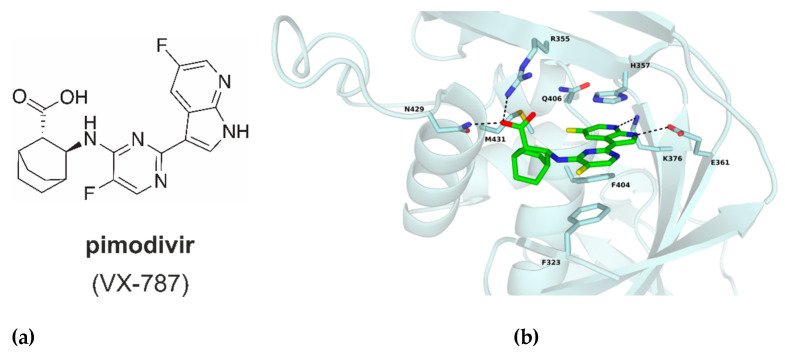
(**a**) Chemical structure of pimodivir (VX-787). (**b**) Cap-binding site of PB2-WT with bound pimodivir (green); ligand, interacting residues and M431 are in stick representation, hydrogen bonds shown as dash lines. PDB code 7AS0 (this manuscript).

One potent inhibitor of the cap-snatching process, pimodivir (VX-787, JNJ-63623872), is a cyclohexyl carboxylic acid analogue inhibitor of host five-prime cap binding to the PB2 subunit (Figure 2a) [8,9]. The binding of pimodivir into the CBS is mediated via four hydrogen bonds with residues N429, R355, K376 and E361. In addition, residues H357, F404 and F323 create π–π stacking with the inhibitor and the azaindole part interacts with residue Q406 (Figure 2b) [9].

During the developmental phase and the clinical trials, several missense mutations were detected within PB2 causing resistance to pimodivir. In preclinical studies, six mutated variants resistant to pimodivir were found–Q306H, S324I, S324N, S324R, F404Y and N541T. For these, the measured EC_50_ (50% effective concentration of pimodivir) were 63- to 257-fold higher in comparison with the wild-type. The most frequently present variant was PB2-F404Y, though all of the mutant variants found within preclinical studies are rare in nature [10]. During the IIa phase of clinical trials, 41 substitutions in PB2 sequence were observed at 38 different positions. Almost every mutated variant occurred only once, but the M431I mutation was found in four volunteer-samples. In this case, 57-fold decrease in sensitivity to pimodivir was measured [11]. In addition to these, Centers for Disease Control and Prevention detected resistant polymorphic mutation PB2-H357N in A/turkey/Minnesota/833/80 (H4N2) [12] while testing nonseasonal influenza viruses from their collection. According to the yet unpublished data, this strain is naturally resistant to pimodivir and mutation H357N conferred approximately 100-fold increase in EC_50_ [13]. All these three residues of PB2 are highly conserved. Mutations F404Y and M431I were selected under drug pressure of pimodivir during clinical studies, and mutation H357N was identified as rare polymorphic [14]. Mutation F404Y did not severely disrupt the viral growth and retained near the wild-type polymerase activity [15]. The examination of M431I substitution revealed a 12.5-fold reduction in replication compared to the wild-type virus [16]. Mutation H357N increased polymerase activity and showed enhanced pathogenic phenotype [14]. Recently, on the 2nd of September 2020, Janssen Pharmaceutical decided to stop Phase III of the clinical development of pimodivir due to insufficient effect that does not bring benefit above currently available standard care. Currently, one compound (CC-42344 from Cocrystal Pharma Inc.) that allegedly binds to the same site of PB2 as pimodivir is in preclinical development stage [17].

We have crystallized proteins of PB2 wild-type (PB2-WT) and selected mutated forms PB2-H357N, PB2-F404Y and PB2-M431I in complex with pimodivir and obtained four high resolution structures. Here we describe the effect of PB2 mutations on pimodivir binding to the PB2 subunit of IAV RdRp and their dissociation constants.

## 2. Results and Discussion

### 2.1. The Mutated Forms M431I, F404Y and H357N of PB2 Impair Its Interaction with Pimodivir

**Figure 3:**
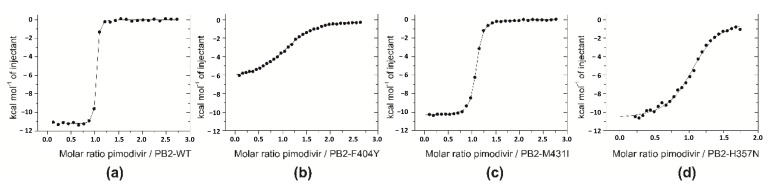
Thermodynamic analyses of pimodivir binding to mutant PB2 cap-binding domains. Isothermal titrations of pimodivir to the cap-binding domain of PB2-WT (**a**), PB2-F404Y (**b**), PB2-M431I (**c**), and PB2-H357N (**d**) performed in 20 mM PIPES, pH 7.5, 150 mM NaCl, 1% DMSO at 25 °C.

We used isothermal titration calorimetry to monitor the binding of pimodivir to wild-type and mutated variants of cap-binding domains of PB2 influenza subunits (Figure 3 and Table 1). Titrations performed in buffers with different enthalpies of ionization (PIPES, Tris or HEPES) yielded the same binding enthalpies indicating that under the experimental conditions there is no net proton transfer coupled to pimodivir binding to PB2 cap-binding domain. Stoichiometries of the complexes were close to 1 (one pimodivir inhibitor to one PB2 cap-binding domain). The dissociation constant (K_d_) of pimodivir binding to wild-type PB2 cap-binding domain was 2.2 nM, which corresponds to a Gibbs energy of binding of −11.8 kcal.mol^−1^. Pimodivir bound to domain with a large favorable enthalpic contribution of −11.1 kcal.mol^−1^ and slightly favorable entropic contribution of -0.8 kcal.mol^−1^. The selected mutations in the cap-binding domains decreased the binding affinity of pimodivir by factors of 280 (F404Y mutant), 7 (M431I mutant) and 130 (H357N mutant).

For the F404Y single mutant, the enthalpy change for pimodivir binding was substantially affected and was 4.8 kcal.mol^−1^ less favorable than the WT value. This change was only partially compensated by a more favorable entropic contribution of 1.4 kcal.mol^−1^ (referenced to the wild-type value).

Compared to WT, the M431I mutant had minor difference in the thermodynamic parameters of pimodivir binding. The inhibitor bound to this mutant with less favorable en-thalpy changes of 0.5 kcal.mol^−1^ and less favorable entropic contribution of 0.6 kcal.mol^−1^.

For the H357N mutant, the enthalpic and entropic contributions were less favorable of 0.5 kcal.mol^−1^ and 2.5 kcal.mol^−1^, respectively.

### 2.2. Crystal Structures Illustrate Influence of Mutations in PB2 on Pimodivir Binding and Help Elucidate the Mechanism of Pimodivir Resistance

**Figure 4:**
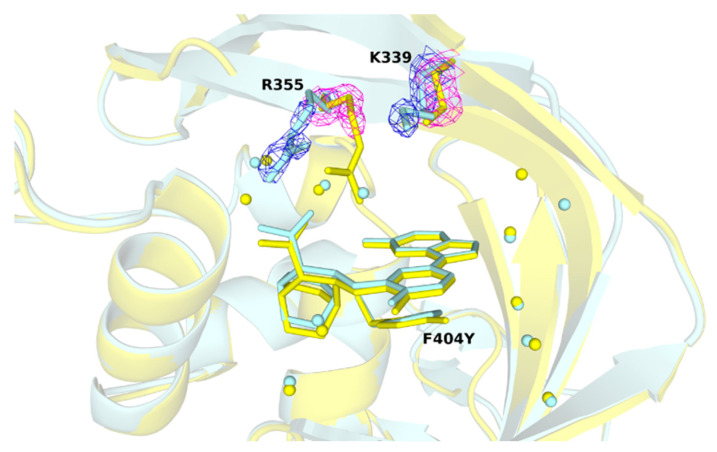
Comparison of the R355 and K339 positions in wild-type and F404Y variant. Bound pimodivir to PB2-WT (pale cyan) and PB2-F404Y (yellow) retain at a close position (RMSD: 0.12 Å). Water molecules are shown as spheres, residue R355 and K339 are shown in stick representation. Details of the 2F_o_–F_c_ electron density maps (both contoured at 1.5 σ) are in blue mesh–for PB2-WT, and magenta mesh for PB2-F404Y.

To find out how mutations of PB2 affect the overall structure, four high resolution crystal structures were compared. Since the thermodynamic analysis showed the greatest loss of the enthalpy contribution in the mutant PB2-F404Y, the structure was compared with that of PB2-WT in detail to identify any visible changes that could explain more unfavorable enthalpy. We observed some poor resolution of two ligand-neighboring amino acids. The uncertainty in positions of K339 and R355 is manifested by higher ADP (atomic displacement parameter) than is the case for other amino acids that form the cap-binding pocket (see Figure 4).

**Figure 5:**
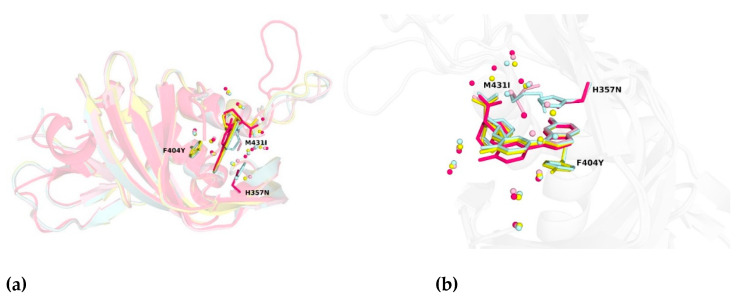
(**a**) Structural alignment of PB2 wild-type and three-point mutation variants in complex with pimodivir. (**b**) Close-up view on the alignment of PB2 crystal structures. Water molecules proximal to the cap-binding site are presented as spheres. The overall structure is depicted as cartoon model with important residues shown as sticks. Color coding is as follows: PB2-WT in pale cyan, PB2-F404Y in yellow, PB2-M431I in light pink, and PB2-H357N in pink.

**Figure 6:**
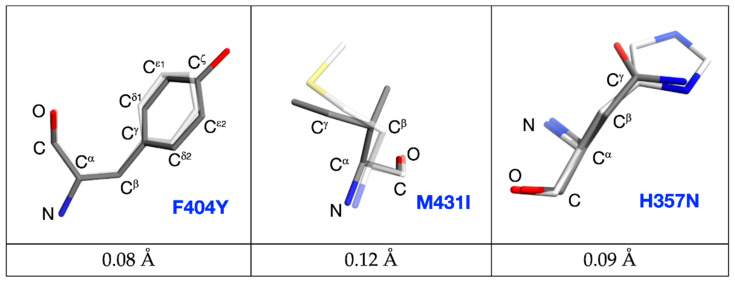
Comparison of the F404-Y404; M431-I431 and H357-N357 positions in wild-type and mutated variants. Wild type PB2 residues are sketched in white-stick representation; mutated forms are in dark-stick representation with common atoms labelled. Root-mean-square deviations calculated for common atoms are listed below.

However, no significant change among the other mutated variants was observed (see Figure 5a). The common atoms of mutated residues F404Y, M431I, and H357N possess similar positions as in the PB2-WT structure. The root-mean-square deviation for PB2-F404Y is 0.08 Å up to C^ζ^. The RMDS of main chain atoms, C^ß^ and C^γ^ for PB2-M431I is 0.12 Å and for PB2-H357N is 0.09 Å. (Figure 6). Phenylalanine/tyrosine 404 and histidine/asparagine 357N create the π–π stacking interaction with pimodivir as well (see Figure 5b). The ligand occupies alike position for each variant as exhibited by the RMSD. The RMSD values for ligands referenced to the PB2-WT are PB2-F404Y–0.12 Å, PB2-M431I–0.16 Å, PB2-H357N–0.29 Å. Therefore, the static X-ray structure analysis does not provide explanation for the differences in binding as observed by calorimetry. Therefore, to explain effects of the mutations on the ligand binding, we have proceeded to the quantum chemistry analysis using solved crystal structures.

### 2.3. Quantum Mechanical Analysis and Subsequent Modelling of PB2 Variants

**Figure 7:**
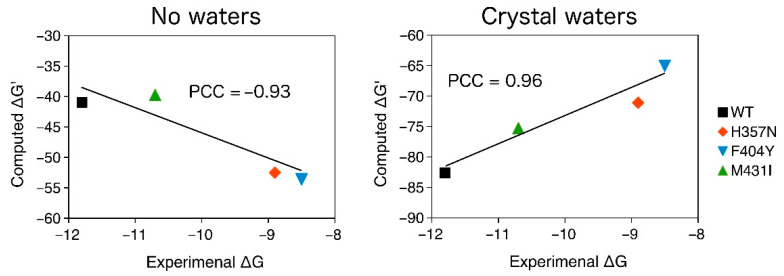
The PM6-D3H4X/COSMO2 binding “free” energies of pimodivir to WT, H357N, M431I, and F404Y variants of PB2 plotted against the experimental binding free energies. The change conformational “free” energy of the protein was not considered. All in kcal.mol^−1^.

The binding of pimodivir to the WT, F404Y, M431I, and H357N variants of PB2 [7,8,9] was examined by using the semiempirical quantum mechanical PM6-D3H4X/COSMO2 based protein ligand scoring function. The correlation between the computed binding “free” energies (scores) and the experimental values are shown in Figure 7. “Free” energies refer to the sums of gas phase energies and solvation free energies. Initially, we omitted the crystal water molecules and the change of protein conformational free energy (∆G’_conf_(P)) in our computational model. Pimodivir had comparable binding affinity to WT and M431I variants of PB2 in such a model. Additionally, the H357N and F404Y mutations considerably increased the binding affinity of the inhibitor. These results disagreed with the experimental observations (Pearson correlation coefficient, PCC of −0.93). The consideration of ∆G’_conf_(P) did not change the results significantly (PCC of −0.83).

In the second step, we extended our computational model by considering the active site water molecules, which considerably improved the correlation with the experimental data (PCC of 0.96). It should be however mentioned that the consideration of ∆G’_conf_(P) worsened the correlation with the experimental data (PCC of 0.84). We will thus further employ the model with the active site water molecules without ∆G’_conf_(P). This model had the highest correlation with the experimental data and correctly ranked WT, M431I, H357N, and F404Y variant of PB2 by binding affinity of pimodivir. The computed binding “free” energies (∆G’), obtained as the sum of interaction energy interaction energy (∆E_int_), interaction solvation free energy (∆∆G_solv_) and change of ligand (L) and (P) conformational “free” energy (∆G’_conf_), are summarized in Table 2.

**Figure 8:**
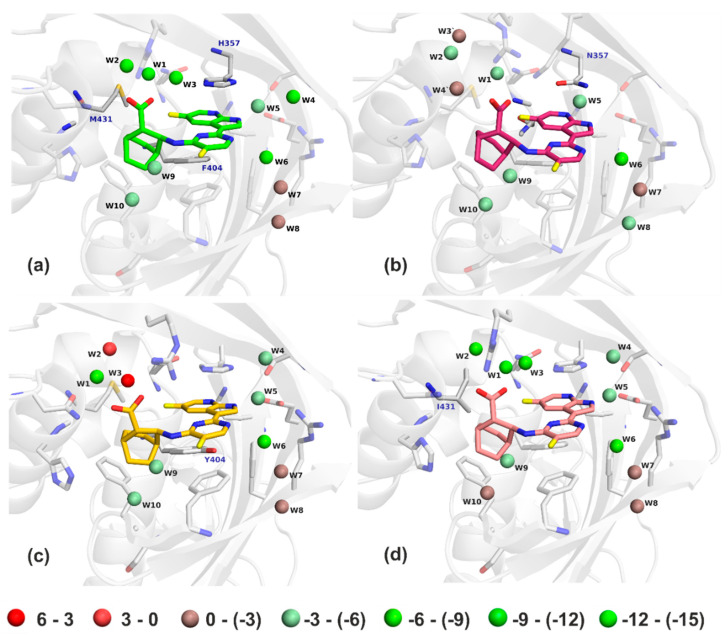
The PM6-D3H4X/COSMO2 “free” energies of bridging water molecules in the WT (**a**), H357N (**b**), F404Y (**c**), and M431I (**d**) structures. Water molecules are colored from red to green (unfavorable to favorable “free” energy, respectively). The energy scale is in kcal.mol^−1^.

A considerably less negative ∆E_int_ of the pimodivir: PB2-F404Y complex can be indicative of the less negative enthalpic term measured by isothermal titration calorimetry (ITC). However, such a comparison requires a caution because part of the enthalpy is included in the interaction solvation free energy term. A detailed analysis of the results revealed that the studied mutations of PB2 affected the binding of pimodivir mainly by changing the “free” energy of the active site water molecules, see Figure 8 and Table 3.

There were ten water molecules bridging pimodivir to the PB2. Positions of the crystallographic and optimized water molecules did not differ significantly (RMSD of 0.9 Å). The largest difference was found for W5 of VX-787/PB2-WT (difference of 1.66 Å). All of the active site water molecules had favorable binding “free” energy in the pimodivir:PB2-WT complex at the PM6-D3H4X/COSMO2 level of theory. Most favorable were water molecules 1 to 6, especially molecules W1 and W2 had highly negative binding “free” energy. The M431I mutation had only a small impact on the “free” energy of the water molecules. W1 and W4 molecules become even more favorable and the other become less favorable (mainly W4, W6, and W10), which resulted in a decrease of the stabilization energy in the pimodivir:PB2-M431I complex. The H357N and F404Y mutations caused a considerable loss of the stabilization “free” energy for water molecules 1 to 4. W2 and W3 became even repulsive upon the F404Y mutation. These changes resulted in a larger decrease in binding affinity of pimodivir to PB2 upon the H357N and F404Y mutations. This might be surprising especially for the F404Y because the residue 404 did not have a direct contact with the mentioned water molecules. The mutation however shifted the position of the inhibitor. Consequently, the conformation of the R355 was changed, which had a large impact on the “free” energy of the water molecules 1 to 3.

Solvent organization is known to be a key contributor to thermodynamics of protein-ligand binding with implications also for drug resistance. It has been reported that a single mutation of the *Haemophilus influenzae* virulence protein SiaP resulted in a 1000-fold drop in sialic acid binding affinity. The mutated amino acid did not have a direct contact with the ligand and the change in affinity was rationalized by an enthalpically unfavorable perturbation of the solvent network [18]. A series of mutations in the active site of Human carbonic anhydrase II was reported to change the thermodynamics parameters of the ligands without direct contact with the ligand and also without changing the protein structure or dynamics. The trends in thermodynamic parameters were rationalized by the modulation of the free energies of active site water molecules [19]. Perturbation of the solvent network also played role in drug resistance mutations in neuraminidase from the 2009 pandemic influenza H1N1 virus [20].

## 3. Materials and Methods

### 3.1. Cloning, Expression, and Purification of Recombinant Proteins

Sequence for PB2-WT cap-binding domain (318-483) from A/California/07/2009 (GenBank FJ96697) was ordered from GenScript Biotech (Piscataway, NJ, USA). The sequence was inserted via *BamHI* and *XhoI* sites into plasmid pETM11-SUMO3 (EMBL, Heidelberg, Germany), which resulted into the plasmid with sequence His_6_-SUMO-PB2. The WT sequence in the plasmid was mutated by PCR mutagenesis to PB2 variants PB2-M431I, PB2-F404Y, and PB2-H357N. All constructs were expressed in *E. coli* BL21 (DE3) RIL. Cells were harvested and resuspended in the lysis buffer (50mM Tris/HCl, pH 8.0, 200mM NaCl, 10mM imidazole). Cell lysate was homogenized and lysed with Emulsiflex device (Avestin, Ottawa, Canada) at a pressure of 1200 bar. Soluble fractions of proteins were purified on Ni-NTA agarose (Roche Diagnostics GmbH, Mannheim, Germany) and eluted with the elution buffer (50mM Tris/HCl, pH 8.0, 200mM NaCl, 250mM imidazole). Subsequent buffer exchange and cleavage of His_6_-SUMO tag by ULP1 protease was completed by overnight dialysis (50mM Tris/HCl, pH 8.0, 200mM NaCl, 1 mM TCEP) at 4 °C. Protein solution was purified by gel permeation chromatography on Superdex 75 (GE Healthcare/Amersham Pharmacia, Uppsala, Sweden). These steps led to more than 95% purity as assessed by SDS-PAGE.

### 3.2. Isothermal Titration Calorimetry

The binding of pimodivir (AdooQ Bioscience, Irvine, CA, USA) to the cap-binding domain of PB2 influenza polymerase subunit was monitored using a VP-ITC microcalorimeter (MicroCal Inc., Malvern Panalytical Ltd., Malvern, UK) at 25 °C in 20 mM PIPES buffer, pH 7.5, containing 150 mM NaCl and 1% DMSO. The exact concentrations of proteins were determined by HPLC amino acid analysis. The inhibitor concentration was determined by elemental analysis. In a 1.43 mL sample cell, protein samples were titrated stepwise with 9 µL injections of inhibitor solution until saturation was achieved. The following protein and inhibitor concentrations were used: 6 µM PB2-WT with 65 µM pimodivir, 8 µM PB2-F404Y with 100 µM pimodivir, 3.4 µM PB2-M431I with 45 µM pimodivir, and 9 µM PB2-H357N with 75 µM pimodivir.

To discern whether inhibitor binding was accompanied by proton transfer, titrations were performed in buffers with different ionization enthalpies (Tris-HCl and HEPES) [21]. All experiments were accompanied by controls in which ligand was injected into buffer alone to determine the dilution heats. Thermodynamic parameters were determined in MicroCal software implemented in Origin 7.0 (MicroCal Inc., Malvern Panalytical Ltd., Malvern, UK).

### 3.3. Protein Crystallization

Purified PB2 variants were dialyzed to the crystallization buffer (10 mM Tris-HCl, pH 8.0, 1 mM TCEP) and concentrated to 2.5 mg/mL (PB2-WT), 3.2 mg/mL (PB2-F404Y), 3.8 mg/mL (PB2-M431I) and 5.2 mg/mL (PB2-H357N) using Amicon 10 kDa centrifugal filter units (MilliporeSigma, Burlington, MA, USA). The proteins were washed with excess solution of 100 μM pimodivir in the crystallization buffer and reconcentrated. Parallelepiped crystals with bound ligand were obtained by the sitting-drop vapor diffusion technique at 18 °C. Three commercial screens were used: Morpheus^®^ (Molecular Dimensions Ltd., Newmarket, UK), SG-1 (Molecular Dimensions Ltd., Newmarket, UK) and JCSG++ (Jena Bioscience, Jena, Germany). Crystallization experiments were set with Oryx8 instrument (Douglas Instruments, East Garston, UK) into 96-well plates (MRC 3 well low, Swissci, Neuheim, Switzerland), where protein to precipitant ratio was 1:1. After several days, crystals of PB2-WT appeared using crystallization reservoir solution: 30 µM sodium fluoride, 30 µM sodium bromide, 30 µM sodium iodide, 20% (*v*/*v*) PEG 500 MME, 10% (*w*/*v*) PEG 20000, 0.1 M imidazole/MES, pH 6.5; for PB2-F404Y and PB2-M431I: 30 µM magnesium chloride, 30 µM calcium chloride, 20% (*v/v*) PEG 500 MME, 10% (*w*/*v*) PEG 20000, 0.1 M bicine/Tris, pH 8.5; and for PB2-H357N: 0.2 M magnesium chloride, 20% (*w*/*v*) PEG 8000, 0.1 M Tris, pH 8.5. Crystals were fished out and flashed-cooled immediately by plunging into liquid nitrogen and stored at −196 °C.

### 3.4. Data Collection and Structure Determination

Diffraction data were collected at −173 °C on a home diffractometer (MicroMax-007 HF microfocus (using Multilyer Optics VariMax^TM^ Cu-VHF Arc)Sec) equipped with a PILATUS 300K detector, Dectris Ltd., Baden-Daettwil, Switzerland). Crystal of PB2-F404Y diffracted up to 1.50 Å; PB2-WT up to 1.55 Å; PB2-H357N up to 1.63 Å and PB2-M431I up to 1.75 Å. Diffraction data were processed using XDS [22] and scaled using XSCALE from XDS suite [23]. The crystals of PB2-WT, PB2-F404Y, PB2-M431I belonged to the P1 space group and all of them contained one molecule in asymmetric unit, with a solvent content of approximately 39%. The crystal of PB2-H357N belonged to the P3_1_21 space group and had also one molecule in asymmetric unit with a solvent content approximately 48%. Crystals’ parameters and data collection statistics are given in Table 4.

Structures of all four PB2 variants were determined by molecular replacement (MOLREP) [24] from the CCP4 package [25], interspersed with manual adjustment in Coot [26], with starting model of a previously published PB2 structure (PDB entry 4P1U, [9]). Refinement was carried out using REFMAC 5.8.0103 [27]. The quality of final models was verified with MolProbity [28]. Refinement statistics are also given in Table 4. All figures of structural representations of PB2 variants were prepared in PyMOL (The PyMOL Molecular Graphics System, Version 1.2r3pre, Schrödinger, LLC., New York, NY, USA) [29]. Atomic coordinates and experimental structure factors have been deposited in the Protein Data Bank under codes 7AS0, 7AS1, 7AS2 and 7AS3.

### 3.5. Structural Analysis of Pimodivir Binding to PB2 Variants

The reported crystal structures of pimodivir with WT, F404Y, M431I, and H357N variants of PB2 were used for molecular modeling. Hydrogen atoms were added to the crystallographic complexes using the AMBER 14 [30], PROPKA 3.0 [31], and Chimera 1.13.1 software [32]. Added hydrogen atoms were relaxed by annealing from 1500 K to 0 K at MM level in AMBER 14. The FF14SB force field was used for the protein while the GAFF force field was used for the ligand [33]. The cooling runs utilized Berendsen thermostat and were 10 ps long with 1 fs steps. Bridging water molecules were considered for all modeled complexes, specifically ten crystal water molecules in all models. It should be mentioned that bromide “Br2” in the PB2-WT/pimodivir crystal structure was considered as a water molecule in the corresponding computational model because Br^-^ ions were not present in the ITC measurements. The position of the inhibitor, water molecules, and residues within 4 Å were optimized in AMBER 14 implying the IGB7 implicit solvent model. The gas-phase interaction energies were computed as the energy difference between the energy of the complex and of its constituent parts (i.e., protein, ligand, and bridging water molecules) [34,35,36] at the PM6-D3H4X [37,38] level of theory using MOPAC2016 [39] and Cuby4 [40,41] software. “Free” energies refer to the sums of gas phase energies and solvation free energies. The solvation free energy was computed using the COSMO2 implicit solvent model [42]. The change of the conformational energy was computed as the “free” energy change between the monomer conformations adopted in the complex and its minimized geometry.

## 4. Conclusions

The purpose of this study was to clarify the yet unknown mechanism of pimodivir resistance development. This inhibitor targets the PB2 subunit, where it binds into the cap binding domain, instead of the five-prime cap. During preclinical and clinical testing of the compound, several point mutations of the viral RNA-dependent RNA polymerase have been identified. Of those, we have selected three major mutations: F404Y, M431I, and H357N that have been described as the most important for the resistance phenotype of the virus. We have used different approaches to reveal the molecular mechanism of the resistance development inferred by these mutations.

Based on the K_D_ determined by ITC, we found that those mutations in the PB2 subunit affect binding affinity of pimodivir. Compared to the wild-type, the dissociation constant changed by the factor of 7 for PB2-M431I, 280 for PB2-F404Y, and 130 for PB2-H357N. This is in accordance with EC_50_ values determined during clinical and preclinical development of the drug. From our four high-resolution structures, the atoms of F404Y, M431I, and H357N possess similar positions as in the PB2-WT. We observed a higher atomic displacement parameter for lysine 339 (K339) and arginine 355 (R355). However, we were not able to sufficiently explain the exact structural mechanism of resistances development from protein structures only. Therefore, we proceeded to molecular modeling and subsequent semiempirical quantum mechanical PM6-D3H4X/COSMO2 “free” energy computations.

The QM/MM analysis showed that the three examined mutations influence the “free” energy of ten water molecules in the cap-binding site. The M431I mutation had only minor influence on the “free” energy of the water molecules. The F404Y and H357N mutations caused a loss of the stabilization “free” energy for water molecules. The mutation F404Y is not in a direct contact with these water molecules. Therefore, the loss of stabilization in this mutant variant could be probably caused by the flexibility of R355. The consequent change of its position might have an impact on the “free energy” of water molecules. Although clinical trials of pimodivir were recently discontinued, this study of resistant mutations against this unique inhibitor will contribute to the development of novel cap-binding domain inhibitors with high barrier towards resistance development.

## Figures and Tables

**Table 1:** Thermodynamic parameters of pimodivir binding to wild-type cap-binding domain of PB2 influenza A polymerase subunit and mutated variants (F404Y, M431I, H357N). Binding values are means and s.d. of two independent experiments performed in 20 mM PIPES (or Tris-HCl), pH 7.5, 150 mM NaCl, 1% DMSO at 25 °C.

	Stoichiometry	ΔG	ΔH	-T.ΔS	K_d_	Fold
PB2 Mutation ^a^	Pimodivir/PB2	kcal.mol^−1^	kcal.mol^−1^	kcal.mol^−1^	nM	K_d_^b^
(wild-type)	0.97 ± 0.04	−11.8 ± 0.1	−11.1 ± 0.3	−0.8 ± 0.4	2.2 ± 0.5	1
F404Y	1.04 ± 0.09	−8.5 ± 0.1	−6.3 ± 0.1	−2.2 ± 0.2	610 ± 100	280
M431I	1.02 ± 0.03	−10.7 ± 0.1	−10.6 ± 0.2	−0.2 ± 0.3	14 ± 1	7
H357N	1.11 ± 0.04	−8.9 ± 0.1	−10.6 ± 0.2	1.7 ± 0.3	290 ± 20	130

^a^ mutations present in cap-binding domain of PB2 ^b^ compared with that of wild-type.

**Table 2:** Computed gas-phase interaction energy (∆E_int_), interaction solvation free energy (∆∆G_solv_), and change of ligand (L) and (P) conformational “free” energy (∆G’_conf_). The binding “free” energy (∆G’) is the sum of ∆E, ∆∆G_solv_ and ∆G’_conf_.

	∆E_int_	∆∆G_solv_	∆G’_conf_(L)	∆G’_conf_(P)	∆G’	∆G’ without ∆G’_conf_(P)
	kcal.mol^−1^	kcal.mol^−1^	kcal.mol^−1^	kcal.mol^−1^	kcal.mol^−1^	kcal.mol^−1^
pimodivir/PB2-WT	−436.3	350.3	3.4	3.9	−78.7	−82.6
pimodivir/PB2-H357N	−445.4	369.8	4.5	0.0	−71.1	−71.1
pimodivir/PB2-F404Y	−394.9	324.4	5.5	8.3	−56.7	−65.0
pimodivir/PB2-M431I	−444.2	365.4	3.5	2.2	−73.1	−75.3

**Table 3:** The PM6-D3H4X binding “free” energies of the active site water molecules. All in kcal.mol^−1^.

Water Molecule	Pimodivir/PB2-WT	Pimodivir/PB2-H357N	Pimodivir/PB2-F404Y	Pimodivir/PB2-M431I
W1	−12.1	−4.5	−8.0	−15.4
W2	−8.3	0.0	2.7	−7.1
W3	−7.6	−3.2	6.1	−8.1
W4	−6.1	−2.2	−3.9	−5.0
W5	−4.4	−5.4	−5.3	−4.3
W6	−7.7	−6.5	−8.6	−6.7
W7	−0.9	−0.7	−1.5	−0.9
W8	−2.6	−4.9	−2.4	−2.3
W9	−3.9	−3.0	−3.3	−3.9
W10	−3.8	−3.8	−3.3	−1.9

**Table 4:** Diffraction data collection and refinement statistics.

PB2 Variant	PB2-WT	PB2-F404Y	PB2-M431I	PB2-H357N
PDB Code	7AS0	7AS1	7AS2	7AS3
**Data Collection Statistics**				
Wavelength (Å)	1.5418	1.5418	1.5418	1.5418
Space group	*P*1	*P*1	*P*1	*P*3_1_21
Cell parameters (Å. ^o^)	29.30, 36.95, 38.34, 71.1, 75.6, 76.3	29.50, 37.25, 38.39, 71.9, 69.8, 75.2	29.19, 37.12, 38.45, 71.9, 75.4, 75.9	64.81, 64.8, 75.63, 90.0, 90.0, 120.0
Resolution range (Å)	50.00–1.55 (1.59–1.55)	50.00–1.50 (1.54–1.50)	50.00–1.75 (1.80–1.75)	50.00–1.55 (1.59–1.55)
Number of unique reflections	20014 (1471)	22637 (1558)	12192 (290)	26694 (1861)
Multiplicity	2.6 (1.8)	2.5 (1.3)	3.0 (1.4)	5.9 (3.9)
Completeness (%)	94.7 (95.3)	98.0 (91.2)	82.6 (26.6)	98.2 (94.1)
R_merge_^a^	5.4 (13.6)	3.5 (9.3)	3.9 (24.9)	4.7 (122)
CC_(1/2)_ (%)	99.7 (96.8)	99.8 (97.6)	99.8 (76.1)	99.9 (46.3)
Average I/σ(I)	12.02 (4.27)	18.27 (4.05)	19.48 (2.61)	17.10 (0.99)
Wilson B (Å^2^)	17.92	21.00	24.20	30.60
**Refinement Statistics**				
Resolution range (Å)	35.64–1.55 (1.59–1.55)	34.93–1.50 (1.54–1.50)	24.34–1.750 (1.79–1.75)	32.42–1.65 (1.69–1.65)
No. of reflection in working set	19032 (996)	21508 (1479)	11584 (274)	21252 (1510)
No. of reflection in the test set	1394 (72)	1129 (77)	610 (14)	1119 (79)
R_work_ value (%) ^b^	0.183 (0.261)	0.150 (0.179)	0.160 (0.236)	0.163 (0.261)
R_free_ value (%) ^c^	0.220 (0.292)	0.185 (0.230)	0.162 (0.286)	0.171 (0.286)
RMSD bond length (Å)	0.02	0.01	0.01	0.01
RMSD angle (^o^)	1.8	1.9	1.6	1.5
Mean ADP value (Å^2^)	13.78	16.85	17.29	25.86
**Ramachandran Plot Statistics ^d^**				
Residues in favored regions (%)	98.12	96.88	96.25	96.45
Residues in allowed regions (%)	1.88	3.12	3.13	3.55

The data in parentheses refer to the highest-resolution shell for data collection statistic. ^a^ R_merge_ = (|I_hkl_ − 〈I〉|)/I_hkl_, where the average intensity 〈I〉 is taken over all symmetry equivalent measurements and I_hkl_ is the measured intensity for any given reflection ^b^ R-value = ||F_o_| − |F_c_||/|F_o_|, where F_o_ and F_c_ are the observed and calculated structure factors, respectively. ^c^ R_free_ is equivalent to R-value but is calculated for 5% of the reflections chosen at random and omitted from the refinement process [27]. ^d^ As determined by Molprobity [28].

## Data Availability

Atomic coordinates and experimental structure factors have been deposited in the Protein Data Bank (http://www.rcsb.org) under codes 7AS0, 7AS1, 7AS2 and 7AS3.
